# Supplementary material for: Validating quality standards in Palestinian emergency departments: An e-Delphi survey approach
Source: PLoS One. 2025 Jan 10;20(1):e0307632. doi: 10.1371/journal.pone.0307632 (PMC11723523; doi:10.1371/journal.pone.0307632)
Supplement: S1 Appendix — (DOCX) [file pone.0307632.s001.docx]

**Validation results of contextual EDQS in Palestine (e-Delphi Survey)**

**Appendix S1: Consensus-based quality standards for emergency departments in Palestine (CBQSEDP) presented for validation and outcome of using two rounds of e- Delphi technique.**

| **Clinical Pathway Domain (A)** | | | |
| --- | --- | --- | --- |
| **Subdomains** | **No. of CBQSEDP** | **The outcome of round 1 e- Delphi** | **The outcome of round 2 e-Delphi** |
| A.1 Triage | 7 | 7 | 7 |
| A.2 Treat or transfer emergency patients | 9 | 9 | 9 |
| A.3 Guidelines, Protocols, and Policies. | 5 | 5 | 5 |
| A.4 Medication Safety | 6 | 6 | 6 |
| A.5 Ambulance Services | 6 | 6 | 6 |
| A.6 Patient flow and length of stay | 3 | 3 | 3 |
| A.7 Medical diagnostic services | 3 | 3 | 3 |
| **Seven Subdomains (A1 – A7)** | **39** | **39** | **39** |
| **Administration Pathway Domain (B)** | | | |
| **Subdomains** | **No. of CBQSEDP** | **The outcome of round 1 e- Delphi** | **The outcome of round 2 e-Delphi** |
| B.1 Documentation and information management system | 7 | 7 | 7 |
| B.2 Access, location, and design | 13 | 13 | 13 |
| B.3 Leadership and management | 5 | 5 | 5 |
| B.4 Workforce staffing and training | 9 | 8 | 8 |
| B.5 Equipment and supplies | 8 | 8 | 8 |
| B.6 Capacity - resuscitation rooms | 5 | 5 | 5 |
| B.7 Resources to support a safe working environment | 7 | 6 | 6 |
| B.8 Performance indicators | 3 | 2 | 2 |
| B.9 Patient safety - infection prevention and control program (PSIPC) | 7 | 7 | 7 |
| **Nine Subdomains (B1 – B9)** | **64** | 61 | 61 |
| **Total of Subdomains = 16** | **103** | **100** | **100** |
