# Supplementary material for: Validating quality standards in Palestinian emergency departments: An e-Delphi survey approach
Source: PLoS One. 2025 Jan 10;20(1):e0307632. doi: 10.1371/journal.pone.0307632 (PMC11723523; doi:10.1371/journal.pone.0307632)
Supplement: S2 Appendix — (DOCX) [file pone.0307632.s002.docx]

**Validation results of contextual EDQS in Palestine (e-Delphi Survey)**

**Appendix S2: Consensus level and modified EDQS after e-Delphi round 1.**

| EDQS - Delphi 1 | Consensus level | Code | Code | Modified EDQS moved to Delphi 2** |
| --- | --- | --- | --- | --- |
| There is an effective evidence-based triage system (process) to prioritize emergency patients (4,5). | 90.9 | A.1.1 | A.1.1 | A triage system is in place to determine priority of healthcare for patients in emergency situations. |
| The hospital has an ambulance to transport patients or there is a permanent transfer agreement with a facility that can provide the required level of care while the patient is being transported (4,5). | 91.8 | A.2.6 | A.2.6 | The emergency department has an ambulance to transport patients or there is a permanent transfer agreement with a facility that can provide the required level of care while the patient is being transported. |
| Ambulance services are under the supervision of the Emergency Department director or the Nursing director (5). | 88.2 | A.5.3 | A.5.3 | The emergency department maintains effective channel of communication ambulance services under the supervision of emergency department director or nursing director. |
| A quality improvement plan and strategies are developed and implemented to minimize the length of stay in the emergency department and improve patient flow in the emergency department based upon an analysis of emergency department processes (4). | 92.7 | A.6.2 | A.6.2 | A quality improvement plan and strategies are developed and implemented to minimize the length of stay and improve patient flow in the emergency department based upon an analysis of emergency department processes. |
| A complete patient record should be kept, including the patient's full name, ID number, arrival date and time, departure date and time, name of ambulance or other form of transport, name(s) of treating medical staff, main complaint and/or diagnosis, and disposition (e.g., home, transfer, admit) of the patient (2,4,5). | 91.8 | B.1.1 | B.1.1 | A complete patient record should be kept, including the patient's full name, unique identifier, arrival and departure date and time, name of ambulance or other form of transport, name(s) of treating medical staff, main complaint, gender, contact information, medical history, allergies, medications, vital signs, physical exam, test results, treatment and interventions, monitoring sheet, informed consent, diagnosis, and disposition of the patient (home, transfer, admit). |
| The registry clerk available all the time to register emergency patients (2). | 95.5 | B.1.2 | B.1.2 | The registry clerk is available all the time to register emergency patients |
| The emergency department has a written policy and procedures for documentation requirements including protection against loss or damage, backup, retention, and alternative systems in case of system failure or crisis (2) | 91.8 | B.1.3 | B.1.3 | The emergency department maintains documented policies and procedures for documentation, safeguards against loss or damage, backup protocols, retention, access control measures, confidentiality, alternative plan in case of system failure or crisis. |
| The emergency department has a written document control policy that includes the preparation, approval, and distribution of documents (2). | 90.9 | B.1.6 | B.1.6 | The emergency department has a written document control policy that includes the preparation, approval, distribution, coding, and change of documents. |
| Emergency department leadership ensure that waiting areas are comfortable (6). | 90.9 | B.2.7 | B.2.7 | Comfortable waiting areas are available in the Emergency Department. |
| There are special places for employees to take a break, change clothes and keep their things (2). | 95.5 | B.2.11 | B.2.11 | There are designated areas within the emergency department where employees can take breaks, change clothes, and store their personal belongings. |
| There is a special place or tent for triage respiratory infections and a special place for isolation (6). | 95.5 | B.2.12 | B.2.12 | There is a dedicated area for triage and isolation respiratory infections. |
| An emergency department manager is a qualified physician by means of education, training, and experience in managing emergency patients (4,5). | 95.5 | B.3.2 | B.3.2 | An emergency department manager is a qualified physician in emergency medicine by means of education, training, and experience. |
| The emergency department is covered by all the required qualified cadres at all shifts and times (4,5). | 94.5 | B.4.1 | B.4.1 | The emergency department is staffed with the necessary qualified personnel during all shifts and hours including doctors, nurses, paramedics, and workers. |
| Emergency department staffing plan is based on historical workload (4,5). | 92.7 | B.4.2 | B.4.2 | Emergency department staffing plan is based on past workload patterns. |
| All Clinical staff in the emergency department must be certified in basic life support (BLS) and advanced cardiovascular life support (ACLS) as appropriate to the ages of the patients served (including Advanced Trauma Life Support) and are available on site or at least one certified individual is assigned on all shifts (2,5). | 93.6 | B.4.7 | B.4.5* | Every Clinical staff working in the emergency department must be certified in basic life support (BLS) and advanced cardiovascular life support (ACLS) as appropriate to the ages of the patients served (including Advanced Trauma Life Support). |
| Infection prevention and control supplies are available around clock, including cleaning materials, disinfectant, liquid soap and personal protective equipment (2). | 95.5 | B.5.8 | B.5.8 | Infection prevention and control supplies are available around the clock, including cleaning materials, disinfectant, liquid soap, and personal protective equipment. |
| In the emergency department there is a room(s) equipped for resuscitation emergencies (4–6). | 95.5 | B.6.1 | B.6.1 | In the emergency department there is a dedicated and equipped room(s) equipped for resuscitation. |
| Security and safety measures are planned and taken to protect emergency department patients and staff (2). | 95.5 | B.7.1 | B.7.1 | Security and safety measures are planned and taken to protect emergency department including patients, staff, and visitors. |
| Adequate and well-trained security personnel are provided to protect emergency department patients and staff (4–6). | 97.3 | B.7.4 | B.7.3* | Adequate and well-trained security personnel are provided to protect emergency department patients, staff, and visitors. |
| Evaluate the effectiveness of the security and safety system for the emergency department on annual basis (4). | 95.5 | B.7.5 | B.7.4* | The security and safety system's effectiveness for the emergency department is assessed annually. |
| Some of the emergency department indicators identified may include, but are not limited to, the following:   - 1. Time to ECG in chest pain patients (5).   2. Time to antibiotics in sepsis patients (5).   3. Triage to physician time (5).   4. Time to enzyme diagnosis (25).   5. patient risk of falls (3).   6. Adequate assessment spaces (17).   7. Reporting system for safety concerns (without fear of reprisal) (17).   8. Analysis of incident reports (17).   9. Sufficient equipment (17).   10. Quality improvement (activity being conducted) (17).   11. Morbidity / Mortality (general or specified conditions) (17).   12. Total length of stay (17).   13. Re admission within 48 hrs. (24) | 89.1 | B.8.3 | B.8.2* | There are various indicators that can be used to measure the performance of emergency departments. Some of these indicators include but are not limited to​​​​​​​​​​​​​​​​​​​​​:   1. Time to ECG in chest pain patients. 2. Time to antibiotics in sepsis patients. 3. Triage to physician time. 4. Time to enzyme diagnosis. 5. patient risk of falls. 6. Adequate assessment spaces. 7. Reporting system for safety concerns (without fear of reprisal). 8. Analysis of incident reports. 9. Sufficient equipment. 10. Quality improvement (activity being conducted). 11. Morbidity / Mortality (general or specified conditions) (17). 12. Total length of stay (17). 13. Re admission within 48 hrs. (24). |
| There is a policies and procedures for most relevant PSIPC issue in emergency department for example, but not limited (Incidence reporting, medication safety, falling down, patient identification, critical result reporting, hand hygiene, waste management, cleaning, disinfection, sterilization, and communication…etc.) (2,6). | 92.7 | B.9.1 | B.9.1 | There are a policies and procedures for most relevant PSIPC issue in emergency department for example, but not limited (Incidence reporting, medication safety, falling down, patient identification, critical result reporting, hand hygiene, waste management, cleaning, disinfection, sterilization, and communication…etc.) |

- * Renumbering the standards, ** Modified standards
