# Supplementary material for: Validating quality standards in Palestinian emergency departments: An e-Delphi survey approach
Source: PLoS One. 2025 Jan 10;20(1):e0307632. doi: 10.1371/journal.pone.0307632 (PMC11723523; doi:10.1371/journal.pone.0307632)
Supplement: S4 Appendix — (DOCX) [file pone.0307632.s004.docx]

**Validation results of contextual EDQS in Palestine (e-Delphi Survey)**

**Appendix S4: EDQS validation results / e-Delphi 1.**

| **Clinical Domain Standards (A) Validation e-Delphi 1 – Data (n=22)** | | | | |
| --- | --- | --- | --- | --- |
| **Subdomain (SD)** | **Standards** | **Mean** | **^a^ Consensus Level %** | **Disagreement level %** |
| **Triage (A.1)** | A.1.1 | 4.55 | 90.9 | 9.1 |
|  | A.1.2 | 4.59 | 91.8 | 8.2 |
|  | A.1.3 | 4.73 | 94.5 | 5.5 |
|  | A.1.4 | 4.68 | 93.6 | 6.4 |
|  | A.1.5 | 4.73 | 94.5 | 5.5 |
|  | A.1.6 | 4.86 | 97.3 | 2.7 |
|  | A.1.7 | 4.59 | 91.8 | 8.2 |
|  | **A.1** |  | **93.5** | **6.5** |
| **Treat or transfer emergency patients (A.2)** | A.2.1 | 4.73 | 94.5 | 5.5 |
|  | A.2.2 | 4.82 | 96.4 | 3.6 |
|  | A.2.3 | 4.68 | 93.6 | 6.4 |
|  | A.2.4 | 4.82 | 96.4 | 3.6 |
|  | A.2.5 | 4.64 | 92.7 | 7.3 |
|  | A.2.6 | 4.59 | 91.8 | 8.2 |
|  | A.2.7 | 4.73 | 94.5 | 5.5 |
|  | A.2.8 | 4.68 | 93.6 | 6.4 |
|  | A.2.9 | 4.73 | 94.5 | 5.5 |
|  | **A2** |  | **94.2** | **5.8** |
| **Guidelines, Protocols and Policies (A.3)** | A.3.1 | 4.50 | 90.0 | 10.0 |
|  | A.3.2 | 4.59 | 91.8 | 8.2 |
|  | A.3.3 | 4.68 | 93.6 | 6.4 |
|  | A.3.4 | 4.77 | 95.5 | 4.5 |
|  | A.3.5 | 4.59 | 91.8 | 8.2 |
|  | **A3** |  | **92.5** | **7.5** |
| **Medication Safety (A.4)** | A.4.1 | 4.82 | 96.4 | 3.6 |
|  | A.4.2 | 4.82 | 96.4 | 3.6 |
|  | A.4.3 | 4.91 | 98.2 | 1.8 |
|  | A.4.4 | 4.59 | 91.8 | 8.2 |
|  | A.4.5 | 4.59 | 91.8 | 8.2 |
|  | A.4.6 | 4.86 | 97.3 | 2.7 |
|  | **A4** |  | **95.3** | **4.7** |
| **Ambulance Service (A.5)** | A.5.1 | 4.73 | 94.5 | 5.5 |
|  | A.5.2 | 4.68 | 93.6 | 6.4 |
|  | A.5.3 | 4.41 | 88.2 | 11.8 |
|  | A.5.4 | 4.73 | 94.5 | 5.5 |
|  | A.5.5 | 4.68 | 93.6 | 6.4 |
|  | A.5.6 | 4.73 | 94.5 | 5.5 |
|  | A5 |  | 93.2 | 6.8 |
| **Patients flow and length of stay (A.6)** | A.6.1 | 4.82 | 96.4 | 3.6 |
|  | A.6.2 | 4.64 | 92.7 | 7.3 |
|  | A.6.3 | 4.77 | 95.5 | 4.5 |
|  | **A6** |  | **94.8** | **5.2** |
| **Medical diagnostic services (A.7)** | A.7.1 | 4.82 | 96.4 | 3.6 |
|  | A.7.2 | 4.77 | 95.5 | 4.5 |
|  | A.7.3 | 4.73 | 94.5 | 5.5 |
|  | A7 |  | 95.5 | 4.5 |
| **Overall Results (A)** | |  | **94.2** | **5.8** |

| **Administration Domain Standards (B) Validation e-Delphi 1 – Data (n=22)** | | | | |
| --- | --- | --- | --- | --- |
| **Subdomain (SD)** | **Standards** | **Mean** | **^a^ Consensus Level** % | **Disagreement level** % |
| **Documentation and Information Management System (B.1)** | B.1.1 | 4.59 | 91.8 | 8.2 |
|  | B.1.2 | 4.77 | 95.5 | 4.5 |
|  | B.1.3 | 4.59 | 91.8 | 8.2 |
|  | B.1.4 | 4.77 | 95.5 | 4.5 |
|  | B.1.5 | 4.77 | 95.5 | 4.5 |
|  | B.1.6 | 4.55 | 90.9 | 9.1 |
|  | B.1.7 | 4.68 | 93.6 | 6.4 |
|  | **B1** |  | **93.5** | **6.5** |
| **Access, location, and design (B.2)** | B.2.1 | 4.86 | 97.3 | 2.7 |
|  | B.2.2 | 4.73 | 94.5 | 5.5 |
|  | B.2.3 | 4.77 | 95.5 | 4.5 |
|  | B.2.4 | 4.77 | 95.5 | 4.5 |
|  | B.2.5 | 4.68 | 93.6 | 6.4 |
|  | B.2.6 | 4.68 | 93.6 | 6.4 |
|  | B.2.7 | 4.55 | 90.9 | 9.1 |
|  | B.2.8 | 4.73 | 94.5 | 5.5 |
|  | B.2.9 | 4.82 | 96.4 | 3.6 |
|  | B.2.10 | 4.73 | 94.5 | 5.5 |
|  | B.2.11 | 4.77 | 95.5 | 4.5 |
|  | B.2.12 | 4.77 | 95.5 | 4.5 |
|  | B.2.13 | 4.64 | 92.7 | 7.3 |
|  | **B2** |  | **94.6** | **5.4** |
| **Leadership and management (B.3)** | B.3.1 | 4.68 | 93.6 | 6.4 |
|  | B.3.2 | 4.77 | 95.5 | 4.5 |
|  | B.3.3 | 4.73 | 94.5 | 5.5 |
|  | B.3.4 | 4.77 | 95.5 | 4.5 |
|  | B.3.5 | 4.73 | 94.5 | 5.5 |
|  | **B3** |  | **94.7** | **5.3** |
| **Workforce staffing and training (B.4)** | B.4.1 | 4.73 | 94.5 | 5.5 |
|  | B.4.2 | 4.64 | 92.7 | 7.3 |
|  | B.4.3 | 4.73 | 94.5 | 5.5 |
|  | B.4.4 | 4.68 | 93.6 | 6.4 |
|  | B.4.5 | 4.77 | 95.5 | 4.5 |
|  | B.4.6 | 4.68 | 93.6 | 6.4 |
|  | B.4.7 | 4.68 | 93.6 | 6.4 |
|  | B.4.8 | 4.50 | 90.0 | 10.0 |
|  | B.4.9 | 4.73 | 94.5 | 5.5 |
|  | **B4** |  | **93.6** | **6.4** |
| **Equipment and Supplies (B.5)** | B.5.1 | 4.77 | 95.5 | 4.5 |
|  | B.5.2 | 4.64 | 92.7 | 7.3 |
|  | B.5.3 | 4.77 | 95.5 | 4.5 |
|  | B.5.4 | 4.82 | 96.4 | 3.6 |
|  | B.5.5 | 4.77 | 95.5 | 4.5 |
|  | B.5.6 | 4.73 | 94.5 | 5.5 |
|  | B.5.7 | 4.73 | 94.5 | 5.5 |
|  | B.5.8 | 4.77 | 95.5 | 4.5 |
|  | **B5** |  | **95.0** | **5.0** |
| **Capacity - Resuscitation rooms (B.6)** | B.6.1 | 4.77 | 95.5 | 4.5 |
|  | B.6.2 | 4.77 | 95.5 | 4.5 |
|  | B.6.3 | 4.73 | 94.5 | 5.5 |
|  | B.6.4 | 4.77 | 95.5 | 4.5 |
|  | B.6.5 | 4.73 | 94.5 | 5.5 |
|  | **B6** |  | **95.1** | **4.9** |
| **Resources to support a safe working environment (B.7)** | B.7.1 | 4.77 | 95.5 | 4.5 |
|  | B.7.2 | 4.68 | 93.6 | 6.4 |
|  | B.7.3 | 4.68 | 93.6 | 6.4 |
|  | B.7.4 | 4.86 | 97.3 | 2.7 |
|  | B.7.5 | 4.77 | 95.5 | 4.5 |
|  | B.7.6 | 4.73 | 94.5 | 5.5 |
|  | B.7.7 | 4.77 | 95.5 | 4.5 |
|  | **B7** |  | **95.1** | **4.9** |
| **Performance Indicators (B.8)** | B.8.1 | 4.82 | 96.4 | 3.6 |
|  | B.8.2 | 4.68 | 93.6 | 6.4 |
|  | B.8.3 | 4.45 | 89.1 | 10.9 |
|  | **B8** |  | **93.0** | **7.0** |
| **Patient Safety - infection prevention and control program (PSIPC) (B.9)** | B.9.1 | 4.64 | 92.7 | 7.3 |
|  | B.9.2 | 4.73 | 94.5 | 5.5 |
|  | B.9.3 | 4.68 | 93.6 | 6.4 |
|  | B.9.4 | 4.59 | 91.8 | 8.2 |
|  | B.9.5 | 4.73 | 94.5 | 5.5 |
|  | B.9.6 | 4.82 | 96.4 | 3.6 |
|  | B.9.7 | 4.73 | 94.5 | 5.5 |
|  | B9 |  | 94.0 | 6.0 |
| **Overall results (B)** | |  | **94.3** | **5.7** |
| **Overall results** | |  | **94.2** |  |

^a^ Threshold of consensus ≥ 80%.
