# Supplementary material for: Validating quality standards in Palestinian emergency departments: An e-Delphi survey approach
Source: PLoS One. 2025 Jan 10;20(1):e0307632. doi: 10.1371/journal.pone.0307632 (PMC11723523; doi:10.1371/journal.pone.0307632)
Supplement: S5 Appendix — (DOCX) [file pone.0307632.s005.docx]

**Validation results of contextual EDQS in Palestine (e-Delphi Survey)**

**Appendix S5: EDQS validation results / e-Delphi 2.**

| **Clinical Domain Standards (A) Validation e-Delphi 2 – Data (n=31)** | | | | | | | | | | | |
| --- | --- | --- | --- | --- | --- | --- | --- | --- | --- | --- | --- |
| **a: Readability Rating** | | | |  | **b: Clarity Rating** | | |  | **c: Compréhensives Rating** | | |
| S.D. | EDQS | Consensus % | Mean |  | EDQS | Consensus % | Mean |  | EDQS | Consensus % | Mean |
| **Triage (A.1)** | A1001a | 98.1 | 4.9 |  | A1001b | 94.8 | 4.7 |  | A1001c | 96.1 | 4.8 |
|  | A1002a | 97.4 | 4.9 |  | A1002b | 96.1 | 4.8 |  | A1002c | 94.8 | 4.7 |
|  | A1003a | 98.1 | 4.9 |  | A1003b | 95.5 | 4.8 |  | A1003c | 94.8 | 4.7 |
|  | A1004a | 96.8 | 4.8 |  | A1004b | 94.2 | 4.7 |  | A1004c | 95.5 | 4.8 |
|  | A1005a | 98.7 | 4.9 |  | A1005b | 97.4 | 4.9 |  | A1005c | 96.1 | 4.8 |
|  | A1006a | 98.7 | 4.9 |  | A1006b | 98.1 | 4.9 |  | A1006c | 98.7 | 4.9 |
|  | A1007a | 95.5 | 4.8 |  | A1007b | 93.5 | 4.7 |  | A1007c | 94.8 | 4.7 |
|  | **A1a** | **97.6** | **4.9** |  | **A1b** | **95.7** | **4.8** |  | **A1c** | **95.9** | **4.8** |
|  | **A1** | **96.4** | **4.8** |  | **A1** | **96.4** | **4.8** |  | **A1** | **96.4** | **4.8** |
| **Treat or transfer emergency patients (A.2)** | A2001a | 96.8 | 4.8 |  | A2001b | 96.8 | 4.8 |  | A2001c | 95.5 | 4.8 |
|  | A2002a | 95.5 | 4.8 |  | A2002b | 95.5 | 4.8 |  | A2002c | 95.5 | 4.8 |
|  | A2003a | 95.5 | 4.8 |  | A2003b | 96.8 | 4.8 |  | A2003c | 95.5 | 4.8 |
|  | A2004a | 97.4 | 4.9 |  | A2004b | 93.5 | 4.7 |  | A2004c | 96.1 | 4.8 |
|  | A2005a | 96.8 | 4.8 |  | A2005b | 95.5 | 4.8 |  | A2005c | 94.8 | 4.7 |
|  | A2006a | 97.4 | 4.9 |  | A2006b | 98.1 | 4.9 |  | A2006c | 96.8 | 4.8 |
|  | A2007a | 98.1 | 4.9 |  | A2007b | 98.1 | 4.9 |  | A2007c | 98.1 | 4.9 |
|  | A2008a | 97.4 | 4.9 |  | A2008b | 96.8 | 4.8 |  | A2008c | 98.1 | 4.9 |
|  | A2009a | 96.1 | 4.8 |  | A2009b | 96.1 | 4.8 |  | A2009c | 94.8 | 4.7 |
|  | **A2a** | **96.8** | **4.8** |  | **A2b** | **96.3** | **4.8** |  | **A2c** | **96.1** | **4.8** |
|  | **A2** | **96.4** | **4.8** |  | **A2** | **96.4** | **4.8** |  | **A2** | **96.4** | **4.8** |
| **Guidelines, Protocols and Policies (A.3)** | A3001a | 94.2 | 4.7 |  | A3001b | 96.1 | 4.8 |  | A3001c | 95.5 | 4.8 |
|  | A3002a | 93.5 | 4.7 |  | A3002b | 92.9 | 4.6 |  | A3002c | 93.5 | 4.7 |
|  | A3003a | 93.5 | 4.7 |  | A3003b | 92.9 | 4.6 |  | A3003c | 92.3 | 4.6 |
|  | A3004a | 96.1 | 4.8 |  | A3004b | 96.1 | 4.8 |  | A3004c | 94.8 | 4.7 |
|  | A3005a | 94.8 | 4.7 |  | A3005b | 92.9 | 4.6 |  | A3005c | 92.9 | 4.6 |
|  | **A3a** | **94.5** | **4.7** |  | **A3b** | **94.2** | **4.7** |  | **A3c** | **93.8** | **4.7** |
|  | **A3** | **94.2** | **4.7** |  | **A3** | **94.2** | **4.7** |  | **A3** | **94.2** | **4.7** |
| **Medication Safety (A.4)** | A4001a | 94.8 | 4.7 |  | A4001b | 96.1 | 4.8 |  | A4001c | 95.5 | 4.8 |
|  | A4002a | 98.1 | 4.9 |  | A4002b | 98.7 | 4.9 |  | A4002c | 98.7 | 4.9 |
|  | A4003a | 95.5 | 4.8 |  | A4003b | 96.8 | 4.8 |  | A4003c | 95.5 | 4.8 |
|  | A4004a | 98.7 | 4.9 |  | A4004b | 96.1 | 4.8 |  | A4004c | 94.8 | 4.7 |
|  | A4005a | 97.4 | 4.9 |  | A4005b | 96.1 | 4.8 |  | A4005c | 96.1 | 4.8 |
|  | A4006a | 98.7 | 4.9 |  | A4006b | 98.1 | 4.9 |  | A4006c | 97.4 | 4.9 |
|  | **A4a** | **97.2** | **4.9** |  | **A4b** | **97.0** | **4.8** |  | **A4c** | **96.3** | **4.8** |
|  | **A4** | **96.8** | **4.8** |  | **A4** | **96.8** | **4.8** |  | **A4** | **96.8** | **4.8** |
| **Ambulance Service (A.5)** | A5001a | 97.4 | 4.9 |  | A5001b | 97.4 | 4.9 |  | A5001c | 98.1 | 4.9 |
|  | A5002a | 97.4 | 4.9 |  | A5002b | 98.1 | 4.9 |  | A5002c | 97.4 | 4.9 |
|  | A5003a | 97.4 | 4.9 |  | A5003b | 96.8 | 4.8 |  | A5003c | 96.8 | 4.8 |
|  | A5004a | 95.5 | 4.8 |  | A5004b | 95.5 | 4.8 |  | A5004c | 95.5 | 4.8 |
|  | A5005a | 96.1 | 4.8 |  | A5005b | 96.1 | 4.8 |  | A5005c | 96.1 | 4.8 |
|  | A5006a | 97.4 | 4.9 |  | A5006b | 96.8 | 4.8 |  | A5006c | 97.4 | 4.9 |
|  | **A5a** | **96.9** | **4.8** |  | **A5b** | **96.8** | **4.8** |  | **A5c** | **96.9** | **4.8** |
|  | **A5** | **96.8** | **4.8** |  | **A5** | **96.8** | **4.8** |  | **A5** | **96.8** | **4.8** |
| **Patients flow and length of stay (A.6)** | A6001a | 97.4 | 4.9 |  | A6001b | 96.8 | 4.8 |  | A6001c | 96.8 | 4.8 |
|  | A6002a | 96.8 | 4.8 |  | A6002b | 96.8 | 4.8 |  | A6002c | 98.1 | 4.9 |
|  | A6003a | 95.5 | 4.8 |  | A6003b | 96.8 | 4.8 |  | A6003c | 97.4 | 4.9 |
|  | **A6a** | **96.6** | **4.8** |  | **A6b** | **96.8** | 4.8 |  | **A6c** | **97.4** | 4.9 |
|  | **A6** | **96.9** | **4.8** |  | **A6** | **96.9** | 4.8 |  | **A6** | **96.9** | 4.8 |
| **Medical diagnostic services (A.7)** | A7001a | 97.4 | 4.9 |  | A7001b | 97.4 | 4.9 |  | A7001c | 97.4 | 4.9 |
|  | A7002a | 97.4 | 4.9 |  | A7002b | 97.4 | 4.9 |  | A7002c | 98.1 | 4.9 |
|  | A7003a | 96.8 | 4.8 |  | A7003b | 96.8 | 4.8 |  | A7003c | 96.8 | 4.8 |
|  | **A7a** | **97.2** | **4.9** |  | **A7b** | **97.2** | **4.9** |  | **A7c** | **97.4** | **4.9** |
|  | **A7** | **97.3** | **4.9** |  | **A7** | **97.3** | **4.9** |  | **A7** | **97.3** | **4.9** |
| **Overall Aa** |  | **96.7** |  | **Overall Ab** |  | **96.3** |  | **Overall Ac** |  | **96.3** |  |
| **Overall A** | **96.4** | | | | | | | | | | |

^a^ Threshold of consensus ≥ 80%.

| **Administration Domain Standards (B) Validation e-Delphi 2 – Data** (n=31) | | | | | | | | | | | |
| --- | --- | --- | --- | --- | --- | --- | --- | --- | --- | --- | --- |
| **a: Readability Rating** | | | |  | **b: Clarity Rating** | | |  | **c: Compréhensives Rating** | | |
| S.D. | EDQS | Consensus % | Mean |  | EDQS | Consensus % | Mean |  | EDQS | Consensus % | Mean |
| **Documentation and Information Management System (B.1)** | B1001a | 96.1 | 4.8 |  | B1001b | 96.8 | 4.8 |  | B1001c | 96.1 | 4.8 |
|  | B1002a | 98.1 | 4.9 |  | B1002b | 98.1 | 4.9 |  | B1002c | 97.4 | 4.9 |
|  | B1003a | 96.1 | 4.8 |  | B1003b | 96.8 | 4.8 |  | B1003c | 95.5 | 4.8 |
|  | B1004a | 97.4 | 4.9 |  | B1004b | 98.1 | 4.9 |  | B1004c | 97.4 | 4.9 |
|  | B1005a | 98.7 | 4.9 |  | B1005b | 98.7 | 4.9 |  | B1005c | 98.7 | 4.9 |
|  | B1006a | 95.5 | 4.8 |  | B1006b | 96.8 | 4.8 |  | B1006c | 94.8 | 4.7 |
|  | B1007a | 98.7 | 4.9 |  | B1007b | 98.7 | 4.9 |  | B1007c | 98.1 | 4.9 |
|  | **B1a** | **97.2** | 4.9 |  | **B1b** | **97.7** | **4.9** |  | **B1c** | **96.9** | **4.8** |
|  | **B1** | **97.3** | 4.9 |  | **B1** | **97.3** | **4.9** |  | **B1** | **97.3** | **4.9** |
| **Access, location, and design (B.2)** | B2001a | 97.4 | 4.9 |  | B2001b | 98.1 | 4.9 |  | B2001c | 97.4 | 4.9 |
|  | B2002a | 98.1 | 4.9 |  | B2002b | 97.4 | 4.9 |  | B2002c | 96.8 | 4.8 |
|  | B2003a | 96.8 | 4.8 |  | B2003b | 96.8 | 4.8 |  | B2003c | 97.4 | 4.9 |
|  | B2004a | 96.1 | 4.8 |  | B2004b | 96.1 | 4.8 |  | B2004c | 97.4 | 4.9 |
|  | B2005a | 96.8 | 4.8 |  | B2005b | 97.4 | 4.9 |  | B2005c | 97.4 | 4.9 |
|  | B2006a | 96.8 | 4.8 |  | B2006b | 96.8 | 4.8 |  | B2006c | 97.4 | 4.9 |
|  | B2007a | 98.1 | 4.9 |  | B2007b | 96.8 | 4.8 |  | B2007c | 97.4 | 4.9 |
|  | B2008a | 98.7 | 4.9 |  | B2008b | 98.1 | 4.9 |  | B2008c | 98.7 | 4.9 |
|  | B2009a | 98.1 | 4.9 |  | B2009b | 96.8 | 4.8 |  | B2009c | 97.4 | 4.9 |
|  | B2010a | 98.7 | 4.9 |  | B2010b | 97.4 | 4.9 |  | B2010c | 99.4 | 5.0 |
|  | B2011a | 98.7 | 4.9 |  | B2011b | 98.1 | 4.9 |  | B2011c | 98.1 | 4.9 |
|  | B2012a | 96.8 | 4.8 |  | B2012b | 97.4 | 4.9 |  | B2012c | 97.4 | 4.9 |
|  | B2013a | 97.4 | 4.9 |  | B2013b | 96.1 | 4.8 |  | B2013c | 96.8 | 4.8 |
|  | **B2a** | **97.6** | 4.9 |  | **B2b** | **97.2** | **4.9** |  | **B2c** | **97.6** | **4.9** |
|  | **B2** | **97.5** | 4.9 |  | **B2** | **97.5** | **4.9** |  | **B2** | **97.5** | **4.9** |
| **Leadership and management (B.3)** | B3001a | 97.4 | 4.9 |  | B3001b | 96.1 | 4.8 |  | B3001c | 96.8 | 4.8 |
|  | B3002a | 98.7 | 4.9 |  | B3002b | 98.1 | 4.9 |  | B3002c | 98.7 | 4.9 |
|  | B3003a | 96.8 | 4.8 |  | B3003b | 96.8 | 4.8 |  | B3003c | 97.4 | 4.9 |
|  | B3004a | 94.8 | 4.7 |  | B3004b | 98.1 | 4.9 |  | B3004c | 96.8 | 4.8 |
|  | B3005a | 96.8 | 4.8 |  | B3005b | 97.4 | 4.9 |  | B3005c | 96.8 | 4.8 |
|  | **B3a** | **96.9** | 4.8 |  | **B3b** | **97.3** | **4.9** |  | **B3c** | **97.3** | **4.9** |
|  | **B3** | **97.2** | 4.9 |  | **B3** | **97.2** | **4.9** |  | **B3** | **97.2** | **4.9** |
| **Workforce staffing and training (B.4)** | B4001a | 95.5 | 4.8 |  | B4001b | 94.8 | 4.7 |  | B4001c | 96.1 | 4.8 |
|  | B4002a | 96.8 | 4.8 |  | B4002b | 96.1 | 4.8 |  | B4002c | 94.8 | 4.7 |
|  | B4003a | 97.4 | 4.9 |  | B4003b | 97.4 | 4.9 |  | B4003c | 97.4 | 4.9 |
|  | B4004a | 97.4 | 4.9 |  | B4004b | 97.4 | 4.9 |  | B4004c | 97.4 | 4.9 |
|  | B4005a | 98.1 | 4.9 |  | B4005b | 98.1 | 4.9 |  | B4005c | 98.7 | 4.9 |
|  | B4006a | 98.1 | 4.9 |  | B4006b | 96.1 | 4.8 |  | B4006c | 98.1 | 4.9 |
|  | B4007a | 98.1 | 4.9 |  | B4007b | 96.8 | 4.8 |  | B4007c | 97.4 | 4.9 |
|  | B4008a | 99.4 | 5.0 |  | B4008b | 98.7 | 4.9 |  | B4008c | 100.0 | 5.0 |
|  | **B4a** | **97.6** | 4.9 |  | **B4b** | **96.9** | **4.8** |  | **B4c** | **97.5** | **4.9** |
|  | **B4** | **97.3** | 4.9 |  | **B4** | **97.3** | **4.9** |  | **B4** | **97.3** | **4.9** |
| **Equipment and Supplies (B.5)** | B5001a | 97.4 | 4.9 |  | B5001b | 97.4 | 4.9 |  | B5001c | 98.1 | 4.9 |
|  | B5002a | 96.1 | 4.8 |  | B5002b | 96.8 | 4.8 |  | B5002c | 96.1 | 4.8 |
|  | B5003a | 96.1 | 4.8 |  | B5003b | 96.8 | 4.8 |  | B5003c | 96.8 | 4.8 |
|  | B5004a | 96.1 | 4.8 |  | B5004b | 96.1 | 4.8 |  | B5004c | 97.4 | 4.9 |
|  | B5005a | 98.1 | 4.9 |  | B5005b | 96.8 | 4.8 |  | B5005c | 96.8 | 4.8 |
|  | B5006a | 97.4 | 4.9 |  | B5006b | 97.4 | 4.9 |  | B5006c | 97.4 | 4.9 |
|  | B5007a | 98.7 | 4.9 |  | B5007b | 97.4 | 4.9 |  | B5007c | 98.1 | 4.9 |
|  | B5008a | 97.4 | 4.9 |  | B5008b | 97.4 | 4.9 |  | B5008c | 97.4 | 4.9 |
|  | **B5a** | **97.2** | 4.9 |  | **B5b** | **97.0** | **4.9** |  | **B5c** | **97.3** | **4.9** |
|  | **B5** | **97.2** | 4.9 |  | **B5** | **97.2** | **4.9** |  | **B5** | **97.2** | **4.9** |
| **Capacity - Resuscitation rooms (B.6)** | B6001a | 98.1 | 4.9 |  | B6001b | 97.4 | 4.9 |  | B6001c | 97.4 | 4.9 |
|  | B6002a | 97.4 | 4.9 |  | B6002b | 98.7 | 4.9 |  | B6002c | 98.1 | 4.9 |
|  | B6003a | 98.7 | 4.9 |  | B6003b | 98.7 | 4.9 |  | B6003c | 98.7 | 4.9 |
|  | B6004a | 98.1 | 4.9 |  | B6004b | 98.7 | 4.9 |  | B6004c | 97.4 | 4.9 |
|  | B6005a | 95.5 | 4.8 |  | B6005b | 96.1 | 4.8 |  | B6005c | 96.1 | 4.8 |
|  | **B6a** | **97.5** | 4.9 |  | **B6b** | **97.9** | **4.9** |  | **B6c** | **97.5** | **4.9** |
|  | **B6** | **97.7** | 4.9 |  | **B6** | **97.7** | **4.9** |  | **B6** | **97.7** | **4.9** |
| **Resources to support a safe working environment (B.7)** | B7001a | 97.4 | 4.9 |  | B7001b | 97.4 | 4.9 |  | B7001c | 97.4 | 4.9 |
|  | B7002a | 96.1 | 4.8 |  | B7002b | 96.8 | 4.8 |  | B7002c | 97.4 | 4.9 |
|  | B7003a | 97.4 | 4.9 |  | B7003b | 96.1 | 4.8 |  | B7003c | 96.8 | 4.8 |
|  | B7004a | 96.8 | 4.8 |  | B7004b | 96.8 | 4.8 |  | B7004c | 96.1 | 4.8 |
|  | B7005a | 96.1 | 4.8 |  | B7005b | 96.8 | 4.8 |  | B7005c | 96.8 | 4.8 |
|  | B7006a | 97.4 | 4.9 |  | B7006b | 96.1 | 4.8 |  | B7006c | 96.8 | 4.8 |
|  | **B7a** | **96.9** | **4.8** |  | **B7b** | **96.7** | **4.8** |  | **B7c** | **96.9** | **4.8** |
|  | **B7** | **96.8** | **4.8** |  | **B7** | **96.8** | **4.8** |  | **B7** | **96.8** | **4.8** |
| **Performance Indicators (B.8)** | B8001a | 96.8 | 4.8 |  | B8001b | 96.8 | 4.8 |  | B8001c | 96.8 | 4.8 |
|  | B8002a | 96.8 | 4.8 |  | B8002b | 95.5 | 4.8 |  | B8002c | 96.1 | 4.8 |
|  | **B8a** | **96.8** | **4.8** |  | **B8b** | **96.1** | **4.8** |  | **B8c** | **96.5** | **4.8** |
|  | **B8** | **96.5** | **4.8** |  | **B8** | **96.5** | **4.8** |  | **B8** | **96.5** | **4.8** |
| **Patient Safety - infection prevention and control program (PSIPC) (B.9)** | B9001a | 98.1 | 4.9 |  | B9001b | 96.8 | 4.8 |  | B9001c | 98.1 | 4.9 |
|  | B9002a | 98.7 | 4.9 |  | B9002b | 98.1 | 4.9 |  | B9002c | 98.7 | 4.9 |
|  | B9003a | 98.7 | 4.9 |  | B9003b | 98.1 | 4.9 |  | B9003c | 98.1 | 4.9 |
|  | B9004a | 98.1 | 4.9 |  | B9004b | 97.4 | 4.9 |  | B9004c | 98.1 | 4.9 |
|  | B9005a | 97.4 | 4.9 |  | B9005b | 98.1 | 4.9 |  | B9005c | 98.1 | 4.9 |
|  | B9006a | 98.7 | 4.9 |  | B9006b | 98.1 | 4.9 |  | B9006c | 97.4 | 4.9 |
|  | B9007a | 98.7 | 4.9 |  | B9007b | 98.1 | 4.9 |  | B9007c | 98.7 | 4.9 |
|  | **B9a** | **98.3** | **4.9** |  | **B9b** | **97.8** | **4.9** |  | **B9c** | **98.2** | **4.9** |
|  | **B9** | **98.1** | **4.9** |  | **B9** | **98.1** | **4.9** |  | **B9** | **98.1** | **4.9** |
| **Overall Ba** |  | **97.3** |  | **Overall Bb** |  | **97.2** |  | **Overall Bc** |  | **97.3** |  |
| **Overall B** | **97.3** | | | | | | | | | | |

^a^ Threshold of consensus ≥ 80%.
