# Supplementary material for: Validating quality standards in Palestinian emergency departments: An e-Delphi survey approach
Source: PLoS One. 2025 Jan 10;20(1):e0307632. doi: 10.1371/journal.pone.0307632 (PMC11723523; doi:10.1371/journal.pone.0307632)
Supplement: S6 Appendix — (DOCX) [file pone.0307632.s006.docx]

**Appendix S6: List of validated emergency departments quality standards (EDQS) – e-Delphi.**

**Triage (A.1)**

**Overview**

The triage process in the emergency department (ED) is a powerful tool for managing emergency patients through a systematic approach and standardized criteria, it identifies and prioritizes people's health problems (1). By triaging patients properly, we are aiming to provide the right treatment at the right time and for the right patient (2). As part of a triage process, trained staff determines which patients require immediate care and how their care is prioritized based on their emergent, urgent, or immediate needs (3), *Table 1.*

| Domain | A | Clinical Pathway |
| --- | --- | --- |
| Subdomain | **A.1** | **Triage** |
| Standards | A.1.1 | A triage system is in place to determine priority of healthcare for patients in emergency situations.  (4,5). |
|  | A.1.2 | Urgent or emergency patients are given priority for timely assessment and appropriate care (5). |
|  | A.1.3 | Patients who have any change in their medical condition or have been waiting for a long period are reassessed periodically (4). |
|  | A.1.4 | The emergency department triage team triages patients using local or international approved guidelines such as Emergency severity index ESI, or WHO triage tool (6). |
|  | A.1.5 | All clinical staff in the triage area must be trained in the triage process and available around the clock (2,6). |
|  | A.1.6 | The triage area should be easily accessible and visible (6). |
|  | A.1.7 | The triage system is implemented in a clear, consistent and non-discriminatory manner for all patients and vulnerable groups (6). |

**Table 1: Triage subdomain and relevant standards.**

**Treat or transfer emergency patients (A.2)**

**Overview**

When the diagnostic and therapeutic interventions required for a patient cannot be found at a given hospital, an inter-hospital transfer (IHT) is necessary (7). There is little knowledge about how the inter-hospital transport team manages critical incidents that occur during inter-hospital transport. In fact, inter-hospital transport is one of the most risky procedures for critically ill patients (8) . It is the transferring hospital's responsibility to provide stabilization treatment and document it within its capacity before transporting the patient (2),*Table 2.*

| Domain | A | Clinical Pathway |
| --- | --- | --- |
| Subdomain | **A.2** | **Treat or transfer emergency patients** |
| Standards | A.2.1 | There is a process in the emergency department to treat or refer emergency patients depending on their needs (4). |
|  | A.2.2 | There is a written policy and procedure for treating emergency patients (4). |
|  | A.2.3 | Emergency patient treatment policy and procedure is implemented and monitored (4). |
|  | A.2.4 | Stabilization therapy is provided to a patient who needs emergency care (4). |
|  | A.2.5 | The emergency department has a written and implemented policy to arrange the patient’s transfer to another hospital, whether the patient or his accompanying person requests the transfer or the hospital has no capacity for treatment (4). |
|  | A.2.6 | The emergency department has an ambulance to transport patients or there is a permanent transfer agreement with a facility that can provide the required level of care while the patient is being transported (4,5). |
|  | A.2.7 | The emergency department team ensures that the patient is informed of the reasons, risks, and benefits of transfer to another hospital. (6). |
|  | A.2.8 | The emergency department team is trained on the policy to prepare patients for transport (6). |
|  | A.2.9 | The emergency department team ensures that all patient referral documents contain sufficient information to facilitate ongoing care when transferring to another hospital (6). |

**Table 2: Treat or transfer emergency patient’s subdomain and relevant standards.**

**Guidelines, Protocols and Policies (A.3)**

**Overview**

Organizations use protocols, guidelines, policies, and procedures to provide uniform knowledge about clinical and nonclinical functions (3). Policies, protocols, and guidelines should be in place in emergency departments to mitigate and equitably share risks to patient safety, and should be updated based on current evidence (6), Table **3**.

| Domain | A | Clinical Pathway |
| --- | --- | --- |
| Subdomain | **A.3** | **Guidelines, Protocols and Policies.** |
| Standards | A.3.1 | For the most common emergencies and the top emergency diagnoses, clinical practice guidelines have been adopted or developed for patient care in emergency departments (4). |
|  | A.3.2 | There are policies and procedures in the emergency department for all clinical and management pathways include but not limited to (5):   - 1. Chest Pain management   2. Management of medico-legal cases such as alcohol and narcotic abuse and criminal acts.   3. Management of suspected victims of abuse, neglect, and domestic violence.   4. Management of suicidal patients   5. Care of trauma patients.   6. Care of patients not competent to care for themselves.   7. Management airway obstruction.   8. Sepsis.   9. Stroke and t-PA.   10. Care of minors (underage).   11. Patient transfer from emergency department to inpatient areas or to another healthcare facility.   12. Patients who leave against medical advice.   13. Patients who leave without being seen. |
|  | A.3.3 | The guidelines, policies and procedures are developed under supervision of emergency department head and in collaboration with qualified relevant staff and departments (5). |
|  | A.3.4 | The emergency department staff are trained on the most common emergencies and the top emergency diagnoses guidelines, policies, and procedures.  (4,5). |
|  | A.3.5 | There is a compliance with the most common emergencies and the top emergency diagnoses emergency department guidelines, policies, and procedures.  (4,5). |

**Table 3: Guidelines, Protocols and Policies subdomain and relevant standards**

**Medication Safety (A.4)**

**Overview**

In hospital environments, such as critical care units and emergency departments, medication errors are reported with alarming frequency. An estimated 4%-14% of medication errors occur in these settings (9). Medication Management is intended to provide an effective and safe system for managing medications, including storage, prescribing, transcribing, preparing, dispensing, and administering (2), *Table* **4**.

| Domain | A | Clinical Pathway |
| --- | --- | --- |
| Subdomain | **A.4** | **Medication Safety** |
| Standards | A.4.1 | There is a policy and procedure for medication management and use in the emergency department (2). |
|  | A.4.2 | The medications are stored safely and securely in the emergency department (6). |
|  | A.4.3 | Life-saving medicines are available in the emergency department at all times and are regularly checked for stock availability, expiration date and storage conditions (2,6). |
|  | A.4.4 | The contents of the crash cart are standardized, well-organized, and inclusive of all necessary medications, including those required for pediatric cases (2). |
|  | A.4.5 | The emergency department has processes in place to ensure that medications are prescribed, administered and recorded correctly and safely (6) |
|  | A.4.6 | Patients in the emergency department are monitored after medications are administered (6). |

**Table 4: Medication safety subdomain and relevant standards**

**Ambulance Service (A.5)**

**Overview**

Ambulance services are important to save lives, and this requires a system that controls this service, with trained staff, adequate equipment, documentation, and effective coordination with emergency departments (7). Transport emergency patients or handling accidents is one of the most important functions of ambulance services in the healthcare system (10). A successful patient transfer depends on the exchange of accurate and complete information between ambulance and hospital personnel (11),

*Table* ***5***.

| Domain | A | Clinical Pathway |
| --- | --- | --- |
| Subdomain | **A.5** | **Ambulance Service** |
| Standards | A.5.1 | Ambulance services are available at all time to transport emergency patients (4). |
|  | A.5.2 | The emergency department has effective channels for cooperation and communication with ambulance services about transferring patients (2,5). |
|  | A.5.3 | The emergency department maintains effective channel of communication ambulance services under the supervision of emergency department director or nursing director (5). |
|  | A.5.4 | Ambulances have necessary equipment and supplies to be ready to transport patients 24/7 (5). |
|  | A.5.5 | All medical, cleaning, disinfection and mechanical requirements of the ambulance are checked and documented on a daily basis (5). |
|  | A.5.6 | Ambulance equipment maintenance is carried out and documented regularly (5). |

***Table 5: Ambulance service subdomain and relevant standards***

**Patients flow and length of stay (A.6)**

**Overview**

Crowding in emergency departments is a significant public health concern (12), Patients in Emergency Departments may experience prolonged waiting times and length of stay (LOS) due to the increasing demand for care and more complex management procedures. The flow of patients through an organization should be seamless, allowing them to move quickly from one episode of care to another (13). Patient flow and staff turnover are both important factors in determining the working conditions of an emergency department, so poor patient flow and high staff turnover may compromise patient safety (14), *Table 6.*

| Domain | A | Clinical Pathway |
| --- | --- | --- |
| Subdomain | **A.6** | **Patients flow and length of stay** |
| Standards | A.6.1 | Patient flow and emergency department processes are monitored and analyzed regularly to reduce length of stay for patients (4) |
|  | A.6.2 | A quality improvement plan and strategies are developed and implemented to minimize the length of stay and improve patient flow in the emergency department based upon an analysis of emergency department processes (4). |
|  | A.6.3 | Patients who need to wait for treatment are informed about waiting times (6). |

**Table 6: Patient flow and length of stay subdomain and relevant standards.**

**Medical diagnostic services (A.7)**

**Overview**

Medical imaging and laboratory tests ordered in the EDs are important factors in patient management. Improved patient outcomes and a shorter length of stay can both be achieved through rapid turnaround of laboratory tests and medical imaging in the ED (15). Diagnostic tests results (such as laboratory, imaging, and cardiac diagnostics) should be communicated in a timely, accurate, complete, unambiguous, and understandable manner reduce errors and improve patient safety (3),

**Table 7***.*

| Domain | A | Clinical Pathway |
| --- | --- | --- |
| Subdomain | **A.7** | **Medical diagnostic services** |
| Standards | A.7.1 | Emergency diagnostic tests (medical imaging and laboratories) are available and performed all the time (5). |
|  | A.7.2 | The results of emergency diagnostic tests are promptly communicated with relevant clinician in a timely manner (5). |
|  | A.7.3 | The emergency department has a process or subcontractor to provide all unavailable emergency diagnostic tests. (5). |

**Table 7: Medical diagnostic services subdomain and relevant standards.**

**Documentation and Information Management System (B.1)**

**Overview**

Medical records accuracy and completeness is both a measure and a means of ensuring the quality of the care that patients receive. Having accurate information in patient medical record can facilitate and enhance communication between healthcare professionals, both within the hospital and when patients are discharged, on the other hand, poor documentation may lead to delays or errors in patient care, especially during care transitions (16), *Table 8.*

| Domain | B | Administration Pathway |
| --- | --- | --- |
| Subdomain | **B.1** | **Documentation and Information Management System** |
| Standards | B.1.1 | A complete patient record should be kept, including the patient's full name, unique identifier, arrival and departure date and time, name of ambulance or other form of transport, name(s) of treating medical staff, main complaint, gender, contact information, medical history, allergies, medications, vital signs, physical exam, test results, treatment and interventions, monitoring sheet, informed consent, diagnosis, and disposition of the patient (home, transfer, admit) (2,4,5). |
|  | B.1.2 | The registry clerk is available all the time to register emergency patients (2). |
|  | B.1.3 | The emergency department maintains documented policies and procedures for documentation, safeguards against loss or damage, backup protocols, retention, access control measures, confidentiality, alternative plan in case of system failure or crisis (2). |
|  | B.1.4 | The emergency department should have a standard list of agreed abbreviations for clinical documentation (2). |
|  | B.1.5 | There should be a process in the laboratory and radiology department to inform the emergency department of critical patient results and document them (2). |
|  | B.1.6 | The emergency department has a written document control policy that includes the preparation, approval, distribution, coding, and change of documents (2). |
|  | B.1.7 | Emergency department staff can access information and data that will support the treatment of patients who present to the emergency department (6). |

**Table 8: Documentation and Information Management System.**

**Access, location, and design (B.2)**

**Overview**

The physical structure of emergency departments is one of the important enabling factors for the quality of the health service, in terms of the size and capacity of the place and the number of rooms that include patient triage, diagnosis, waiting and reception, in addition to the appropriate environment in terms of hygiene, clean running water, staff facilities, ventilation and lighting, as the appropriate design helps in the flow of patients and staff comfortably It prevents overcrowding in emergency departments. In addition, easy access to the entrance to the emergency department and places of diagnostic service such as laboratories and medical imaging (17), ***Table 9****.*

| Domain | B | Administration Pathway |
| --- | --- | --- |
| subdomain | **B.2** | **Access, location, and design** |
| Standards | B.2.1 | The Location of emergency department is easily accessible, visible and clearly identifiable. (4,6) |
|  | B.2.2 | The emergency entrance is defined by visible signs and easy access, whether by ambulance or car, and patients (4,6). |
|  | B.2.3 | Patients can get in and out the emergency department without going through other areas of the hospital (4,6). |
|  | B.2.4 | The entrance to the emergency department is suitable for patients with special needs and is equipped with the necessary tools for them (4,6). |
|  | B.2.5 | The emergency department layout allows easy access to equipment and resources by the emergency department team. (6) |
|  | B.2.6 | The emergency department has safe ways to evacuate in case of emergency (6). |
|  | B.2.7 | Comfortable waiting areas are available in the Emergency Department (6). |
|  | B.2.8 | The emergency department ensures availability of running water for cleaning, hand washing and drinking around the clock (2). |
|  | B.2.9 | The emergency department ensures that electricity is available 24 hrs. a day, every day (2). |
|  | B.2.10 | There should be adequate toilets for emergency department users with privacy for collecting samples, especially urine and stool (2) |
|  | B.2.11 | There are designated areas within the emergency department where employees can take breaks, change clothes, and store their personal belongings (2). |
|  | B.2.12 | There is a dedicated area for triage and isolation respiratory infections (6). |
|  | B.2.13 | There must be at least 1 ED bed per 5000 annual patient visits (18). |

**Table 9: Access, location, and design subdomain and relevant standards.**

**Leadership and management (B.3)**

**Overview**

Emergency departments must have their own organizational structure, whether they are part of the hospital or independent, and be responsible for providing the service and ensuring its quality and safety, according to the regulations and laws. Proper leadership of emergency departments invests in available resources, and provides an effective team committed to continuous improvement and able to serve the emergency patient based on quality and safety standards (2,6,19), Table 10.

| Domain | B | Administration Pathway |
| --- | --- | --- |
| Subdomain | **B.3** | **Leadership and management** |
| Standards | B.3.1 | Responsibilities and duties are defined by approved job description for key leadership functions in the emergency department (2). |
|  | B.3.2 | An emergency department manager is a qualified physician in emergency medicine by means of education, training, and experience (4,5). |
|  | B.3.3 | The emergency department manager oversee the development and implementation of all policies and procedures related to managing emergency patients (4,5). |
|  | B.3.4 | The emergency department nursing manager is a qualified nurse by education, training, and experience specifically in emergency patient care (5). |
|  | B.3.5 | The emergency department nursing manager oversee the development and implementation of all nursing policies and procedures related to managing emergency patients (4,5). |

**Table 10: Leadership and management subdomain and relevant standards.**

**Workforce staffing and training (B.4)**

**Overview**

*“The heart and soul of any organization is its people”* (20). It is important for emergency departments to have competent, multi-skilled, well-trained, qualified, and motivated staff to deliver efficient, effective, and timely patient centered care, compliant with local or international requirements regarding staffing numbers, including nurses, doctors, and other professionals, as well as teamwork and communication between the departments (2,17,20), Table 11*.*

| Domain | B | Administration Pathway |
| --- | --- | --- |
| Subdomain | **B.4** | **Workforce staffing and training** |
| Standards | B.4.1 | The emergency department is staffed with the necessary qualified personnel during all shifts and hours including doctors, nurses, paramedics and workers (4,5). |
|  | B.4.2 | Emergency department staffing plan is based on past workload patterns (4,5). |
|  | B.4.3 | There is an approved policy and procedure on how consultants are called for their opinions (4,5). |
|  | B.4.4 | There is a plan to provide additional staff in case of overload (patients overflow) (4). |
|  | B.4.5 | Every Clinical staff working in the emergency department must be certified in basic life support (BLS) and advanced cardiovascular life support (ACLS) as appropriate to the ages of the patients served (including Advanced Trauma Life Support) (2,5). |
|  | B.4.6 | The non-clinical support staff must be trained in CPR (2). |
|  | B.4.7 | Emergency department staff receive continuous education with competency assessment (5). |
|  | B.4.8 | Certified clinical staff with BLS, ACLS, and advanced trauma life support must be covered all shifts or at least one designated per shift (2,5). |
|  |  |  |

**Table 11: Workforce staffing and training subdomain and relevant standards.**

**Equipment and Supplies (B.5)**

**Overview**

Having access to equipment, tools, and consumables around the clock is one of the most essential resources for providing safe, effective, and sustainable health care in emergency departments. Staff training and effective maintenance programmes are essential for maintaining the functionality of these devices (2,6,17), Table 12*.*

| Domain | B | Administration Pathway |
| --- | --- | --- |
| Subdomain | **B.5** | **Equipment and Supplies** |
| Standards | B.5.1 | There is an up-to-date list of all functioning equipment and supplies needed for the emergency department (2,6). |
|  | B.5.2 | The emergency department equipment and supplies are available in sufficient quantity, proper for special needs, tidy and well organized (2,6). |
|  | B.5.3 | There is an effective equipment maintenance program in place for the emergency department (2,6). |
|  | B.5.4 | There are spare equipment and tools in case they break down (2). |
|  | B.5.5 | The emergency department staff are trained to use the available equipment (2,6). |
|  | B.5.6 | The resuscitation / trauma rooms have all the necessary equipment and supplies, and their functioning is checked daily on all shifts (5,6). |
|  | B.5.7 | There are a documented procedures in place to ensure the availability of medical gases around the clock to include procurement, safe handling, storage, distribution, regular inspection, use and renewal (2,5). |
|  | B.5.8 | Infection prevention and control supplies are available around clock, including cleaning materials, disinfectant, liquid soap and personal protective equipment (2). |

**Table 12: Equipment and supplies subdomain and relevant standards.**

**Capacity - Resuscitation rooms (B.6)**

**Overview**

Each Emergency Department should have an adequate Resuscitation Room, for an ED with an average case mix of 20,000 patients per year, two resuscitation bays are recommended, with one bay per 10,000 patients. In the design of resuscitation rooms, radiation safety measures should be taken into consideration, and the room should be easily accessible and close to the ambulance entrance, as well as the environment of the place should be suitable in terms of climate and lighting (18), *Table 13***.**

| Domain | B | Administration Pathway |
| --- | --- | --- |
| Subdomain | **B.6** | **Capacity - Resuscitation rooms** |
| Standards | B.6.1 | In the emergency department there is a dedicated and equipped room(s) equipped for resuscitation (4–6). |
|  | B.6.2 | Resuscitation / trauma rooms are suitable and have sufficient space to perform resuscitation (4–6). |
|  | B.6.3 | All essential resuscitation medications are available (4–6). |
|  | B.6.4 | The emergency department has policies and procedures to deal with cases that need resuscitation (4). |
|  | B.6.5 | The emergency department has a plan to respond to resuscitation cases at any time and any place in the hospital and includes at least (4):   1. Define the team, its responsibilities, and the way of communications. 2. Training the team of resuscitation. 3. Emergency medicines, where they are located, and how to secure them. 4. Required equipment and ensuring its effectiveness. 5. Evaluate the effectiveness of the plan periodically |

**Table 13: Capacity - Resuscitation room’s subdomain and relevant standards.**

**Resources to support a safe working environment (B.7)**

**Overview**

Throughout the world and in Palestine, workplace violence against healthcare workers (HCWs) in emergency departments is a major threat to their workplace safety and health. The victims, patients, and healthcare organizations all suffer significant consequences as a result (21,22). To safeguard everyone in the hospital against personal harm and loss or damage to property, a written security program should be implemented effectively (3), Table 14.

| Domain | B | Administration Pathway |
| --- | --- | --- |
| Subdomain | **B.7** | **Resources to support a safe working environment** |
| Standards | B.7.1 | Security and safety measures are planned and taken to protect emergency department including patients, staff, and visitors (2). |
|  | B.7.2 | The emergency department has a safety and security plan based on identified safety and security threats, for example, natural and manmade disasters, mass causality management, and evacuation plan (2). |
|  | B.7.3 | Adequate and well trained security personnel are provided to protect emergency department patients, staff, and visitors (4–6). |
|  | B.7.4 | The security and safety system's effectiveness for the emergency department is assessed annually (4). |
|  | B.7.5 | There is a warning mechanism in place to alert the security team if any security issue occurs (2,4). |
|  | B.7.6 | The emergency department posts safety warning signs in a language that patients, families, and community members understand (2). |
|  |  |  |

**Table 14: Resources to support a safe working environment subdomain and relevant standards.**

**Performance Indicators (B.8)**

**Overview**

Quality assessment of emergency department is very important for continuous improvement (23). The purpose of performance indicators monitoring is to determine whether the emergency department services meets its goals and standards through the collection of data (24), Table 15.

| Domain | B | Administration Pathway |
| --- | --- | --- |
| Subdomain | **B.8** | **Performance Indicators** |
| Standards | B.8.1 | Clinical and managerial quality indicators in relation to the emergency department's structure, processes, and outcomes,  are defined, measured and reported regularly (2). |
|  | B.8.2 | There are various indicators that can be used to measure the performance of emergency departments. Some of these indicators include, but are not limited to​​​​​​​​​​​​​​​​​​​​​:   - 1. Time to ECG in chest pain patients (5).   2. Time to antibiotics in sepsis patients (5).   3. Triage to physician time (5).   4. Time to enzyme diagnosis (25).   5. patient risk of falls (3).   6. Adequate assessment spaces (17).   7. Reporting system for safety concerns (without fear of reprisal) (17).   8. Analysis of incident reports (17).   9. Sufficient equipment (17).   10. Quality improvement (activity being conducted) (17).   11. Morbidity / Mortality (general or specified conditions) (17).   12. Total length of stay (17).   13. Re admission within 48 hrs. (24). |
|  |  |  |

**Table 15: Performance indicators subdomain and relevant standards.**

**Patient Safety - infection prevention and control program (PSIPC) (B.9)**

**Overview**

The commitment to patient safety is both a human right and an important component of quality in healthcare, as well as a concept that covers all processes within the healthcare system (26,27). The studies found that around 10% of all inpatient admissions result in some level of unintended harm to the patient. Up to 75% of these lapses are preventable (27). Therefore, it is necessary to have an effective program to ensure patient safety including IPC in emergency departments. The leadership of emergency departments is responsible for developing, implementing, monitoring and controlling strategies for preventing, managing, and controlling infections and antimicrobial resistance; reducing harm to patients, visitors, staff, and visitors; and achieving good patient outcomes (19), Table 16.

| Domain | B | Administration Pathway |
| --- | --- | --- |
| Subdomain | **B.9** | **Patient Safety - infection prevention and control program (PSIPC)** |
| Standards | B.9.1 | There are a policies and procedures for most relevant PSIPC issue in emergency department for example, but not limited (Incidence reporting, medication safety, falling down, patient identification, critical result reporting, hand hygiene, waste management, cleaning, disinfection, sterilization, and communication…etc.) (2,6). |
|  | B.9.2 | The emergency department staff are trained on the PSIPC policies and procedures (2,6). |
|  | B.9.3 | All policies and procedures of PSIPC are implemented and monitored regularly (2,6). |
|  | B.9.4 | Patient safety attitude are measured regularly among the staff of emergency department (6). |
|  | B.9.5 | All morbidity and mortality in the emergency department are reviewed by a multidisciplinary team on a regular basis (2). |
|  | B.9.6 | A regular audit is conducted in the emergency department for continuous improvement (2). |
|  | B.9.7 | A proactive risk assessment is conducted in the emergency department and mitigation actions are taken (2). |

**Table 16: Patient Safety - infection prevention and control program (PSIPC) subdomain and relevant standards.**

**References**

1. Bambi S, Ruggeri M, Sansolino S, Gabellieri M, Tellini S, Giusti M, et al. Emergency department triage performance timing. A regional multicenter descriptive study in Italy. Int Emerg Nurs [Internet]. 2016;29:32–7. Available from: http://dx.doi.org/10.1016/j.ienj.2015.10.005

2. Quality & Accreditation Institute center for accreditation of health & SC. Accreditation Standards for Emergency Department [Internet]. 1st ed. Quality and Accreditation Institute; 2021. 153 p. Available from: http://www.qai.org.in/ALLDOC/doc00000196.pdf

3. JCI. Joint Commission International accreditation standards for hospitals : including standards for Academic Medical Center Hospitals. [Internet]. Joint Commission Resource; 2021. 391 p. Available from: https://www.jcrinc.com/

4. HCAC. Health Care Accreditation Council Hospital Accreditation Standards 4 th EDITION, 2 nd Version Effective 2020 STD HCAC Hospital Accreditation Standards 4 th Edition, 2 nd Version. 2020.

5. Saudi Central Board for Accreditation of Healthcare institutions. CBAHI Standards | CBAHI National Hospital Standard 3rd Edition [Internet]. 2015 [cited 2021 Oct 13]. Available from: https://portal.cbahi.gov.sa/english/cbahi-standards

6. ACEM. Quality Standards for EMERGENCY DEPARTMENTS and other HOSPITAL-BASED EMERGENCY CARE SERVICES [Internet]. 2015 [cited 2021 Oct 13]. Available from: www.acem.org.au

7. Sethi D, Subramanian S. When place and time matter: How to conduct safe inter-hospital transfer of patients. Saudi J Anaesth. 2014;8(1):104–13.

8. Bergman L, Pettersson M, Chaboyer W, Carlström E, Ringdal M. Improving quality and safety during intrahospital transport of critically ill patients: A critical incident study. Aust Crit Care. 2020;33(1):12–9.

9. Di Simone E, Giannetta N, Auddino F, Cicotto A, Grilli D, Di Muzio M. Medication errors in the emergency department: Knowledge, attitude, behavior, and training needs of nurses. Indian J Crit Care Med. 2018;22(5):346–52.

10. Dúason S, Gunnarsson B, Svavarsdóttir MH. Patient handover between ambulance crew and healthcare professionals in Icelandic emergency departments: a qualitative study. Scand J Trauma Resusc Emerg Med. 2021;29(1):1–11.

11. Sanjuan-Quiles Á, Hernández-Ramón MDP, Juliá-Sanchis R, García-Aracil N, Castejón-De La Encina ME, Perpiñá-Galvañ J. Handover of Patients from Prehospital Emergency Services to Emergency Departments: A Qualitative Analysis Based on Experiences of Nurses. J Nurs Care Qual. 2019;34(2):169–74.

12. Kusumawati HI, Magarey J, Rasmussen P. Analysis of factors influencing length of stay in the Emergency Department in public hospital, Yogyakarta, Indonesia. Australas Emerg Care. 2019 Sep 1;22(3):174–9.

13. Haybarker BD. ScholarWorks Reducing Emergency Department Length of Stay by System Change. 2015; Available from: https://scholarworks.waldenu.edu/dissertations

14. Burström L. Patient Safety in the Emergency Department: Culture, Waiting, and Outcomes of Efficiency and Quality [Internet]. 2014. Available from: http://www.diva-portal.org/smash/record.jsf?pid=diva2:714718

15. Kaushik N, Khangulov VS, O’hara M, Arnaout R. Reduction in laboratory turnaround time decreases emergency room length of stay. Open Access Emerg Med. 2018;10:37–45.

16. Lorenzetti DL, Quan H, Lucyk K, Cunningham C, Hennessy D, Jiang J, et al. Strategies for improving physician documentation in the emergency department: A systematic review 11 Medical and Health Sciences 1117 Public Health and Health Services. BMC Emerg Med. 2018;18(1).

17. Hansen K, Boyle A, Holroyd B, Phillips G, Benger J, Chartier LB, et al. Updated framework on quality and safety in emergency medicine. Emerg Med J. 2020;37(7):437–42.

18. Irish Association for Emergency Medicine. Standards for Emergency Department Design and Specification for Ireland. 2007;(September):57. Available from: http://www.iaem.ie/wp-content/uploads/2013/02/iaem_standards_for_ed_design__specification_for_ireland_300907.pdf

19. Australian Commission on Safety and Quality in Health Care. National Safety and Quality Health Service Standards. 2nd ed. [Internet]. 2021. 1–92 p. Available from: www.safetyandquality.gov.au

20. Alnajem M, Garza-Reyes JA, Antony J. Lean readiness within emergency departments: a conceptual framework. Benchmarking. 2019;26(6):1874–904.

21. D’Ettorre G, Mazzotta M, Pellicani V, Vullo A. Preventing and managing workplace violence against healthcare workers in emergency departments. Acta Biomed. 2018;89(1):28–36.

22. Hamdan M, Abu Hamra A. Workplace violence towards workers in the emergency departments of Palestinian hospitals: A cross-sectional study. Hum Resour Health. 2015;13(1):1–9.

23. Broccoli MC, Moresky R, Dixon, Julia et al. Defining quality indicators for emergency care delivery: findings of an expert consensus process by emergency care practitioners in Africa. BMJ Glob Heal. 2018;3(1):e000479.

24. Wakai A, O’Sullivan R, Staunton P et al. Development of key performance indicators for emergency departments in Ireland using an electronic modified-Delphi consensus approach. Eur J Emerg Med. 2013 Apr;20(2):109–14.

25. Graff L, Stevens C, Spaite D, Foody JA. Measuring and improving quality in emergency medicine. Acad Emerg Med. 2002;9(11):1091–107.

26. Källberg A-S. Patient Safety in the Emergency Department – Errors, Interruptions and Staff Experience. 2015. 1–58 p.

27. WHO. Patient safety assessment manual [Internet]. World Health Organization. 2011. 1–230 p. Available from: http://www.who.int/patientsafety/research/emro_afro_report.pdf
