# Supplementary material for: Validating quality standards in Palestinian emergency departments: An e-Delphi survey approach
Source: PLoS One. 2025 Jan 10;20(1):e0307632. doi: 10.1371/journal.pone.0307632 (PMC11723523; doi:10.1371/journal.pone.0307632)
Supplement: Attachment — Submitted filename: Response to reviewers.DOCX [file pone.0307632.s007.DOCX]

**Response to Reviewers**

**Manuscript Title**: Validating Quality Standards in Palestinian Emergency Departments: An e-Delphi Survey Approach

**Manuscript ID:** PONE-D-24-25666

**Date:** 27/11/2024

**Dear Naeem Mubarak,**

**Academic Editor / PLOS ONE**

**Dear Reviewers,**

We sincerely thank you for your time and constructive feedback, which has significantly enhanced the quality of our manuscript. Below, we provide detailed responses to each comment raised by the editor and reviewers. All revisions are highlighted (track changes) in the manuscript for your convenience.

**Responses to Editorial Comments**

**Comment 1: PLOS ONE's style requirements.**

**Response:** The manuscript has been revised to meet the PLOS style requirements.

**Comment 2: Please include a complete copy of the PLOS questionnaire on inclusivity in global research in your revised manuscript.**

**Response:** We have included the full text of the PLOS inclusivity questionnaire as Supporting Information (S1 Attachment) in the manuscript.

**Comment 3: Please provide additional details regarding participant consent.**

**Response:** Additional details have been added to the methods section under the subheading *Delphi Panel Recruitment and Sample* (Page 8). The study did not involve minors. These details have also been added to the “Ethics Statement” field of the submission form.

**Comment 4: Please include captions for your Supporting Information files at the end of your manuscript and update any in-text citations accordingly.**

**Response:** Captions for all Supporting Information files have been added to the end of the manuscript. Additionally, all in-text citations have been updated to match these files, ensuring consistency.

**Comment 5: Please review your reference list to ensure that it is complete and correct.**

**Response:** The reference list has been reviewed and confirmed to be complete and accurate.

**Responses to Reviewer #1 Comments**

**Comment 1: Introduction – Elaboration on previous shortcomings in the literature.**

**Response:** A paragraph has been added to the introduction on Page 5 / line 127, discussing conventional methods and the approach utilized in this study.

**Comment 2: Methods – Delphi panel recruitment and sample.**

**Response:** Measures to mitigate potential bias during Delphi rounds have been detailed under the *Delphi Panel Recruitment and Sample* section on Page 7 / line 168. Additionally, participants were allowed to complete the survey in stages using the Lime Survey platform to ensure thoughtful responses. Summarized the mitigation action as follows:

(Measures were taken at several stages to mitigate potential bias related to survey length. **First,** Previous efforts aimed to streamline standards by identifying appropriate ones for the Palestinian context through literature review and input from local experts, enhancing the clarity of the standards and aligning them with the study objectives. **Second**, Participants received clear instructions and guidance before starting the survey, including its purpose and estimated completion time. This transparency helped manage expectations and encouraged thoughtful responses. **Additionally,** reminders were sent during the survey period to encourage participation without exerting pressure, which could have compromised the quality of responses. **Finally,** the survey design through the Lime Survey platform allowed for completing the survey in several stages and not at the same time, as the participant can save the answers at the last point and complete it at another time if he feels bored, tired, or busy).

**Comment 3: Methods – Readability, clarity, and comprehensiveness.**

**Response:** A short paragraph is added within the methods section/study instrument/on page 8 / line 199.

**Comment 4: Methods – Round 1 evaluation and shortlisting.**

**Response:** The paragraph under *Round 1 Evaluation and Shortlisting* (Page 9 / line 219) has been rephrased, with added elaboration on the interquartile range (IQR).

**Comment 5: Discussion – Literature comparison.**

**Response:** The second paragraph of the discussion on (Page 14 / lines 331-340) has been rewritten to include a comparison with existing literature and explain the contribution of these criteria to ongoing improvements.

**Comment 6: Literature Review – Examples of standards.**

**Response:** Examples of standards from prior studies and literature have been included in the introduction on (Page 4/ lines 107 -118).

**Comment 7: Formatting adjustments.**

- Inclusion of line numbers: **Resolved.**
- Consistent page numbering format: **Resolved.**
- Renaming "Background" to "Introduction": **Resolved.**

**Responses to Reviewer #2 Comments**

**Comment 1: The abstract is too long.**

**Response:** The abstract has been shortened, particularly the methodology and conclusion sections.

**Comment 2: Provide more details about Palestine's previously derived EDQS in the introduction.**

**Response:** Additional details have been added on Page 7, Lines 138–149.

**Comment 3: Trauma quality standards should have priority.**

**Response:** These EDQS are comprehensive, covering all emergency services, including standards specific to trauma (e.g., equipment, resuscitation room, ACLS/BLS training). Details are provided in Appendix S 5.

**Comment 4: 103 quality standards for Palestinian EDs are too many; fewer would be better.**

**Response:** The final list comprises 100 validated EDQS. While comprehensive, the standards are divided into manageable domains, subdomains, and standards. Future research may explore their application to identify areas for consolidation.

We thank the reviewers and editor again for their valuable comments. Please do not hesitate to contact me if further clarification is needed. We look forward to your feedback.

**Sincerely,**
Abed Alra'oof Bani Odeh

UCT – PhD candidate

27/11/2024
